# Students' satisfaction with simulated clinical experiences: validation of an assessment scale

**DOI:** 10.1590/0104-1169.3295.2471

**Published:** 2014

**Authors:** Rui Carlos Negrão Baptista, José Carlos Amado Martins, Maria Fátima Carneiro Ribeiro Pereira, Alessandra Mazzo

**Affiliations:** 1 Doctoral student, Instituto de Ciências Biomédicas Abel Salazar, Universidade do Porto, Porto, Portugal. Adjunct Professor, Unidade Científico-Pedagógica de Enfermagem Médico-Cirúrgica, Escola Superior de Enfermagem de Coimbra, Coimbra, Portugal; 2 PhD, Coordinator Professor, Unidade Científico-Pedagógica de Enfermagem Médico-Cirúrgica, Escola Superior de Enfermagem de Coimbra, Coimbra, Portugal; 3 PhD, Assistant Professor, Faculdade de Psicologia e de Ciências da Educação, Universidade do Porto, Porto, Portugal; 4 PhD, Professor, Escola de Enfermagem de Ribeirão Preto, Universidade de São Paulo, WHO Collaborating Centre for Nursing Research Development, Ribeirão Preto, SP, Brazil

**Keywords:** Personal Satisfaction, Students, Nursing, Simulation

## Abstract

**OBJECTIVE:**

validate an assessment instrument of nursing students' satisfaction with simulated clinical experiences.

**METHOD:**

a 17-item scale was applied to students from the Teaching Diploma Program in Nursing, after a set of simulated clinical experiences. Factorial analysis with orthogonal varimax rotation was used, and the internal consistency was estimated to determine the validity of the scale.

**RESULTS:**

in a sample of 181 students, we found a high correlation between practically all items and the total scale, with an Alpha coefficient of 0.914. The scale items were divided in three factors: practical dimension, realism dimension and cognitive dimension, with good internal consistency coefficients of 0.89; 0.88 and 0.73, respectively.

**CONCLUSION:**

the scale complies with the validity requisites, revealing a high potential for use in research.

## Introduction

The construction of nursing knowledge and the way it is transmitted to the students have evolved over the years, permitting the progressive construction of a scientific corpus that sustains nursing as a science^(^ 1 ^)^.

The evolution of science in general and of technology itself, associated with the growing health requirements of society lead to increasing pressure in nursing schools and their teachers, with a view to the development of more and better prepared professionals^(^ 2 ^)^.

Nursing faculty and teachers are confronted with challenges that drive them to create new pedagogical approaches that promote the students' self-discovery and stimulate their active search in the development of learning itself^(^ 3 ^)^.

Thus, nursing is experiencing the use of new active teaching methods. The study of clinical cases, the creation of clinical scenarios, laboratory practice and simulation are examples, with particular interest and highlight, whether because of their specific teaching characteristics or the benefits they offer for the students' education.

These changes in the teaching/education paradigms currently put the students at the heart of their learning, in which they are the engine of their development. As a result, traditional teaching methods like lectures and oral presentations are considered less appropriate to develop some kinds of learning^(^ 3 ^)^.

Scientific evidence in the field of simulation demonstrates that, when used in teaching, it enhances and promotes the development of significant learning in the students, and that it can reach its culmination if the participants consider it as legitimate, authentic and realistic^(^ 2 ^)^. Furthermore, there is strong evidence that the students appreciate the simulation and the opportunities created to practice it in a safe and risk-free environment^(^ 4 ^)^.

The results of simulation in health education are more beneficial when associated with the most modern sound and image technologies, realistic environments, high-fidelity simulators and a structured reflection after each scenario about the learning and the decisions taken (debriefing), core concepts to talk about a simulated clinical experience^(^ 1 ^)^.

Various authors have analyzed the students' satisfaction with the simulation^(^ 5 ^-^ 13 ^)^, although the results of the satisfaction scores obtained in the different groups of students who used different simulators did not all converge. These contradictory results and the fact that none of them represented the Portuguese reality stimulates the development of further research in the area and theme.

Students' satisfaction is an important result as it is associated with greater involvement in the process and greater motivation for learning.

Hence, it seemed pertinent to elaborate an instrument that would allow us to assess the nursing students' satisfaction with simulated practice in a Portuguese reality. Therefore, the objective in this study is to validate a scale of satisfaction with the simulated clinical experiences.

## Methods

### Study subjects

In a population of 318 fourth and final-year students from the Teaching Diploma Program in Nursing, all of whom complied with the eligibility criteria, 181 students were included in the sample. Having participated in the practical classes on emergency nursing and accepting to participate in the study were established as the inclusion criteria.

### Data collection process

In the fourth year, as part of the Curricular Unit Emergency Nursing, with an hour load of 18 theoretical-practical hours and 18 practical hours, the students apply the specific competences gained in earlier years and develop a set of simulated clinical experiences, based on the solution of complete and complex scenarios. Besides the development of competences to act in emergency situations, teamwork, leadership, problem solving in complex environments, decision making and assertive communication are aimed for, among others.

The scenarios take place at a Simulation Center, with a realistic environment, material and equipment and using medium (Advanced Life Support Mannequins Megacod^(r)^ adult and Junior, with VitalSim^(r)^, by Laerdal^(r))^ and high-fidelity (iStan^(r)^ (adult) and PediaSim^(r)^ (Junior) by Meti^(r))^ patient simulators.

At the end of this Curricular Unit, the students were invited to participate in the study and received information about its objectives and the anonymous and voluntary nature of their participation.

### Ethical considerations

The study received authorization from the School Board and a favorable opinion from the Ethics Committee of the Health-Nursing Sciences Research Unit at *Escola Superior de Enfermagem de Coimbra* (P 03-10/2010). A written consent form was used.

### Construction process of the scale

To construct the scale, two previously developed studies were important, one with a phenomenological approach, addressing the students' experiences in simulated practice using high-fidelity manikins, in which 13 intentionally selected informants, in an interview with one open question (How did you experience the simulated practice with high-fidelity manikins?) and five guiding questions, manifested what they felt after the simulated practice; and the other a systematic literature review about the gains the students perceived after simulated practices, in which various theme areas emerged, including satisfaction.

Thus, these studies resulted in a list of 17 items, which we called the *Escala de Satisfação com as Experiências Clínicas Simuladas* (ESECS). The students express their opinion about the 17 assertions on a Likert scale, ranging from one to ten, in which one represents the lowest and ten the highest level of satisfaction.

### Data analysis

After collecting all questionnaires, a database was elaborated in the software Statistical Package for the Social Sciences (SPSS, version 18 for Windows), through which we used descriptive statistics with central trend and dispersion measures (mean, mode, median, percentiles, variance, standard deviation) to characterize the sample and the statistical inference (factorial analysis and estimated internal consistency) to determine the validity and reliability of the scale. To assess the obtained results, a p-value <0.05 was set as statistically significant.

For the purpose of this study and to guarantee more robust results, we considered ten participants for each item of the scale that is to be analyzed^(^ 14 ^)^.

## Results

### Sample

The sample consists of 181 fourth-year students from the Teaching Diploma Program in Nursing, who in April 2012 volunteered to participate in the study, on the final class day of the curricular unit on Emergency Nursing.

Most participants (76.80%) are female (Table 1). The subjects' ages varied between 20 and 32 years, with a larger percentage in the age group between 21 and 23 years (87.29%), a mean age of 22.11 years and a standard deviation of 1.90 years.

**Table 1:** - Distribution of the students' sociodemographic characteristics (N=181)

Variables		N	%
Course year			
	4^th^ year	181	100,00
Age group			
	< 21 years	1	0,55
	21 – 23 years	158	87,29
	24 – 26 years	13	7,18
	27 – 29 years	6	3,32
	30 – 32 years	3	1,66
Gender			
	Male	42	23,20
	Female	139	76,80

### Validity of the items and reliability of the scale

First, it was analyzed whether the set of items in the ESECS is related with satisfaction by submitting the proposed items to the Cronbach's Alpha test (Table 2) and determine their correlation. A high correlation coefficient was found between practically all items and the total scale, which resulted in a high Alpha coefficient (0.914). In addition, all items contribute to a good Alpha coefficient, and eliminating any of them would negatively affect the scale, except for the item "motivation to attend practical classes", which maintained the global Alpha coefficient*.*

**Table 2:** - Homogeneity statistics of items and Cronbach's internal consistency coefficients of the global ESECS (N=181)

Items	Mean	Standard deviation	Item-total correlation (corrected)	Alpha if the item were eliminated
Global satisfaction with practical classes	8.558	1.029	0.685	0.907
Learning achieved	8.320	0.854	0.603	0.909
Motivation to attend practical classes	8.082	1.440	0.482	0.914
Dynamics of practical classes	8.939	0.989	0.597	0.909
Active participation in the scenarios developed	7.939	1.256	0.624	0.908
Interaction with colleagues	8.292	0.992	0.651	0.908
Interaction with teachers	8.375	1.065	0.669	0.907
Satisfaction with scenarios’ level of difficulty	8.342	1.112	0.630	0.908
Satisfaction with debriefing	8.745	1.183	0.523	0.911
Link between scenarios and theory	9.099	1.150	0.543	0.910
Appropriateness to themes developed in TP classes	8.745	1.317	0.483	0.913
Productivity during practical classes	8.331	1.169	0.704	0.906
Realism of the scenarios developed	8.834	1.056	0.675	0.907
Credibility during the scenario	8.320	1.158	0.695	0.906
Quality of the material used in the practicums	8.861	1.158	0.530	0.911
Quality of the equipment used in the practicums	8.939	1.080	0.547	0.910
Quality of the simulators	9.138	0.929	0.557	0.910

### Construct validity

For the construct validity, the factor analysis of the main components of the correlations among the variables was used to summarize as much of the information as possible in as few factors as possible.

The Kaiser-Meyer-Olkin measure revealed that the sample was appropriate for the analysis, with a coefficient of 0.874. Bartlett's sphericity test revealed statistically significant results with X^2 ^= 2033.842 at *p *<0.001, which indicated that a relation exists among the variables that are to be included and, thus, the factor analysis is considered appropriate. The observation of the Scree Plot shows a clear division into three factors, which explain 63.80% of the variance, positioned before the inflection and tending to rectify from that point onwards. In view of the sample size, the convergence of the Scree Plot and the Kaiser criterion, this number of factors was maintained in the final analysis.

The main components analysis was followed by the orthogonal varimax rotation of the data, applying the Kaiser normalization and thus reducing the number of variables with high loadings per factor.

After defining the three factors, the factor loadings for each item were verified, excluding those with factor loading less than 0.45 (Table 3). In addition, the rational coherence of the proposed solution was verified, guaranteeing a conceptual translation of the mathematical proposal.

**Table 3:** - Saturation matrix of items in the factors for the orthogonal Varimax solution with Kaiser's normalization for three factors (N=181)

Items	Factors		
	1	2	3
Global satisfaction with practical classes	0.811		
Learning achieved	0.747		
Motivation to attend practical classes	0.800		
Dynamics of practical classes	0.554		
Active participation in the scenarios developed	0.742		
Interaction with colleagues	0.576		
Interaction with teachers	0.644		
Satisfaction with scenarios’ level of difficulty	0.578		
Satisfaction with debriefing			0,482
Link between scenarios and theory			0,883
Appropriateness to themes developed in TP classes			0,843
Productivity during practical classes	0.651		
Realism of the scenarios developed		0.569	
Credibility during the scenario	0.466	0.537	
Quality of the material used in the practicums		0.935	
Quality of the equipment used in the practicums		0.930	
Quality of the simulators		0.839	

Eigenvalues less than 0.45 were omitted

Factor 1 explains 28.31% of the total variance and consists of nine items (1,2,3,4,5,6,7,8 and 12) related to the "practical dimension", whether individually, in group or interacting with the teacher. The factor loadings ranged from 0.811 (global satisfaction with practices) as the highest value to 0.554 (dynamics of practical classes) as the lowest.

Factor 2 explains 20.57% of the variance and consists of five items (13,14,15,16 and 17) related to the "realism dimension", because of the scenarios' approximation of the real context with the manikins' physiological response to the student's action. The factor loading varies between 0.935 and 0.537, related to the quality of the material used in the practices and the credibility during the scenario, respectively. Item 14 (credibility during the scenario) saturates with similar values in factors 1 and 2 (0.466 and 0.537), but the researchers decided to include it in factor 2, considering not only the statistical result, with a higher saturation coefficient, but a theoretical coherence based on scientific knowledge about simulated practice as significant learning, provided that the students consider it as legitimate, authentic and realistic^(^ 2 ^)^.

Factor 3 explains a variance of 14.90% and consists of three items (9, 10 and 11), related to the "cognitive dimension", manifested by the post-simulated practice reflections to complement and internalize what was taught in the classroom as well. The highest saturation in this factor corresponds to the link between the scenarios and the theory (0.883) and the lowest to the satisfaction with the debriefing (0.482).

### Internal consistency of the scale dimensions

After analyzing the internal consistency coefficient of the ESECS as a whole, it is coherent to analyze each of the dimensions separately in the same manner. We found high correlation coefficients in all items, with values exceeding 0.60, except in the practical dimension for items 4 (0.581) and 8 (0.591) and in the cognitive dimension for item 9, with an item-total correlation coefficient of 0.374. The Alpha coefficients of each dimension remain high (factor 1: 0.891; factor 2: 0.888; factor 3: 0.736), indicating good internal consistency (Table 4).

**Table 4:** - Item-total correlation coefficients of each scale dimension and respective internal consistency coefficients (N=181)

Dimensions and respective items		Correlation with total (corrected)	Cronbach’s Alpha
Satisfaction with practical dimension (9 items)			0,891
	Global satisfaction with practical classes	0.756	
	Learning achieved	0.659	
	Motivation to attend practical classes	0.602	
	Dynamics of practical classes	0.581	
	Active participation in the scenarios developed	0.709	
	Interaction with colleagues	0.641	
	Interaction with teachers	0.688	
	Satisfaction with scenarios’ level of difficulty	0.591	
	Productivity during practical classes	0.682	
Satisfaction with realism dimension (5 items)			0.888
	Realism of the scenarios developed	0.677	
	Credibility during the scenario	0.645	
	Quality of the material used in the practicums	0.805	
	Quality of the equipment used in the practicums	0.793	
	Quality of the simulators	0.740	
Satisfaction with cognitive dimension (3 items)			0.736
	Satisfaction with debriefing	0.374	
	Link between scenarios and theory	0.723	
	Appropriateness to themes developed in TP classes	0.618	
Global coefficient of the Scale			0.914

### Descriptive results of the ESECS

Based on the analysis of Table 5 and the central trend and dispersion measures of the global scale and each of its dimensions, the students are highly satisfied with the simulated practice. The highest mean coefficients were found for the cognitive dimension (88.63%), although this dimension showed the lowest minimum value (50.00%), while practical satisfaction revealed the lowest mean values (83.53%). In all dimensions, the maximum coefficient was 100.00%.

**Table 5:** - Descriptive statistics of each dimension and the global scale

		Satisfactionpractical	Satisfaction realism	Satisfaction cognitive	Satisfaction global
Mean		83.53	88.18	88.63	85.80
Median		83.33	90.00	90.00	86.47
Mode		80.00*	100.00	100.00	84.71
Standard Deviation		8.13	8.97	9.86	7.28
Variance		66.16	80.52	97.26	53.05
Minimum		51.11	64.00	50.00	64.71
Maximum		100.00	100.00	100.00	100.00
Percentiles					
	25	78.88	82.00	83.33	81.17
	50	83.33	90.00	90.00	86.47
	75	88.88	96.00	96.66	90.88

* As the dimension was multimodal, the lowest coefficient was displayed.

In the dispersion of the satisfaction, visible in the percentile distribution, it is verified that more than 75% of the sample displays practical satisfaction levels superior to 78.88%, and levels over 80% for the other two dimensions and the global scale.

## Discussion

In Portugal, simulated practice in nursing teaching is a reality in a significant part of the curricular units offered within the school walls, where the students feel they are developing the competencies to be able to face the clinical context with greater self-confidence, autonomy and satisfaction.

It is true that high levels of satisfaction with the simulated practice do not always translate a good clinical performance, but the students' satisfaction is a good measure to evaluate the teaching, the teachers and the university itself. It promotes qualitative improvements in teaching, where the student's opinion as a "client" and beneficiary of a service is increasingly taken into consideration. The important link between satisfaction and motivation to learn is well-known, which is of particular interest in the current generation of students, who are flooded with a wide range of stimuli. Thus, a motivated student learns more and better, believing in the potential utility of what (s)he is learning for future practice.

As there does not exist any scale in Portugal to measure the nursing students' satisfaction with the simulated practice, a scale with 17 assertions was constructed and applied in a population of students who were concluding the curricular unit on emergency nursing, with a view to the refining and factorial validity of each item.

The database used attended to the proposed objectives, with 10 observations for each variable under analysis, which permits guarantees robust and credible results. The Bartlett test coefficients and Kaiser-Meyer-Olkin measure showed that the sample was appropriate for the factor analysis of the items^(^ 14 ^)^.

The Varimax orthogonal rotation was chosen to make the empirical result more easily interpretable, without affecting the statistical properties^(^ 15 ^)^. To interpret the value of each variable in the definition of each factor, a correlation between the variable and the factor >0.45 was assumed as the minimum acceptable value.

In the validation process, a high correlation coefficient was verified between practically all items and the total scale, with a good reliability ratio (Alpha = 0,914), indicating that the scale seems to measure the students' satisfaction with the simulated practice. Based on statistical support to guarantee the appropriateness of the factor analysis, a clear division in three factors stood out, with good reliability ratios for each isolated factor, close to 0.90.

The students' satisfaction level with the practice in a simulated context is high, which stimulates the school to invest in this teaching strategy, always looking for the best results.

The students appreciated more the realism and the cognitive satisfaction. The lower coefficients in the practical dimensions seem to be related with the insufficient time dedicated to the practical component. Even this variation can be considered as a validity criterion of the scale though, due its ability to distinguish among different dimensions of the concept.

## Conclusion

In this study, we aimed to present the results of the validation process of a scale to assess the nursing students' satisfaction with the simulated clinical experiences developed in the academic context.

The ESECS shows high reliability and validity coefficients, which allow us to affirm that it has the potential to analyze/assess the nursing students' satisfaction with the simulated practice. Its application in students at other teaching levels, in different nursing colleges and different courses where the practical components are a teaching/learning strategy is pertinent. Therefore, the current results should be interpreted with some caution.

Another score in favor of the ESECS' reliability is related to the agreement with the proposed factorial division, deriving from the mathematical analysis and its coherent rational meaning.

The scale has good conceptual and psychometric properties. It is considered a simple instrument that is easy to answer, which contributes to its application in future studies. Nevertheless, this study comes with some limitations, such as the small sample size and the sole focus on the satisfaction with simulated practices using medium and high-fidelity manikins and in the specific context of emergency nursing, which should be broadened to the entire practice in the laboratory context.

Future studies can contribute to reinforce the reliability of the ESECS as a research instrument.

## References

[1] Martins JC, Mazzo A, Baptista RCN, Coutinho VRD, Godoy S, Mendes IAC (2012). The simulated clinical experience in nursing education:
A historical review. Acta Paul Enferm..

[2] Leigh GT (2008). High-Fidelity Patient Simulation and Nursing Students
Self-Efficacy: a review of the literature. Int J Nurs Educ Scholarsh..

[3] Hawkins K, Todd M, Manz J (2008). A Unique Simulation Teaching Method. J Nurs Educ..

[4] Dillard N, Sideras S, Ryan M, Carlton KH, Lasater K, Siktberg L (2009). A Collaborative Project to Apply and Evaluate the
Clinical Judgment Model Through Simulation. Nurs Educ Perspect..

[5] Jeffries PR, Rizzolo MA (2006). Designing and Implementing Models for the Innovative Use of
Simulation to Teach Nursing Care of Ill adults and Children: A national,
multi-site, multi-method study.

[6] Hoadley TA (2009). Learning Advanced Cardiac Life Support: a comparison
study of the effects of low and high-fidelity simulation. Nurs Educ Perspect..

[7] Kardong-Edgren S, Lungstrum N, Bendel R (2009). VitalSim versus SimMan: A comparison of BSN student test
scores, knowledge retention, and satisfaction. Clin Simul Nurs..

[8] Zulkosky KD (2010). Simulation use in the classroom: Impact on knowledge
acquisition, satisfaction, and self-confidence. Clin Simul Nurs..

[9] Kuznar KA (2007). Associate degree nursing students' perceptions of
learning using a high-fidelity human patient simulator. Teach Learn Nurs..

[10] Smith SJ, Roehrs CJ (2009). Hight-Fidelity Simulation: Factors Correlated with
Nursing Student Satisfaction and Self-Confidence. Nurs Educ Perspect..

[11] Butler KW, Veltre DE, Brady DS (2009). Implementation of Active Learning Pedagogy Comparing
Low-Fidelity Simulation Versus High-Fidelity Simulation in Pediatric Nursing
Education. Clin Simul Nurs..

[12] Reilly A, Spratt C (2007). The perceptions of undergraduate student nurses of
high-fidelity simulation-based learning: A case report from the University of
Tasmania. Nurse Educ Today..

[13] Swenty CF, Eggleston BM (2010). The Evaluation of Simulation in a Baccalaureate Nursing
Program. Clin Simul Nurs..

[14] Hair JF Jr, Black WC, Babin BJ, Anderson RE (2010). Multivariate Data Analysis.

[15] Beavers AS, Lounsbury JW, Richards JK, Huck SW, Skolits GJ, Esquivel SL (2013). Practical Considerations for Using Exploratory Factor
Analysis in Educational Research. Pract Assess Res Eval. [Internet].

